# *p53*, *Cyclin-D1*, *β-catenin*, *APC* and *c-myc* in Tumor Tissue from Colorectal and Gastric Cancer Patients with Suspected Lynch Syndrome by the Bethesda Criteria 

**DOI:** 10.31557/APJCP.2020.21.2.343

**Published:** 2020

**Authors:** Tais Fernanda Marcolino, Celia Aparecida Marques Pimenta, Ricardo Artigiani Neto, Paula Castelo, Marcelo Souza Silva, Nora Manoukian Forones, Celina Tizuko Fujiyama Oshima

**Affiliations:** 1 *Oncology Group, Gastroenterology Division, Department of Medicine, *; 2 *Department of Pathology, Federal University of Sao Paulo, Sao Paulo, Brazil. *

**Keywords:** Colorectal cancer, gastric cancer, Lynch syndrome, immunohistochemistry

## Abstract

Colorectal cancer (CRC) is one of the most frequent neoplasms worldwide, and up to 15% have a family history. Lynch syndrome (LS) is a hereditary cause of CRC and gastric (GC). Individuals with LS have mutations in mismatch genes repair. p53, cyclin D1, β-catenin, APC and c-myc proteins are involved in the cell cycle and carcinogenesis.

**Objective:**

To study the expression of p53, Cyclin D1, β-catenin, APC and c-myc proteins in patients with CRC and GC with at least one of the Bethesda positive criteria. Compare the expression of these proteins with the presence or absence of expression of the DNA repair proteins.

**Patients and Methods:**

We included 70 individuals with CRC or GC with at least one of the Bethesda positive criteria. Protein expression of MLH1, MSH2, MSH6, PMS2, p53, cyclin D1, β-catenin, APC and c-myc were analized by immunohistochemistry tumours tissues.

**Results:**

Deficient expression of MLH1, MSH2, MSH6 and PMS2 were respectively 38.7%; 17.7%; 26.22% and 48.38%. We found a negative association between deficiency of PMS2 and age, and positive association between PMS2 deficiency and APC positive. The positive imunoexpression of APC increases by 4 times the chance of having deficiency of PMS2.

**Conclusions:**

Patients with loss of expression of *PMS2* had a higher risk of mutation or deletion of APC and tumours with positive immunoexpression of *cyclin D1* had an increased risk of loss of expression of *MSH2*. These results suggest that tumours with loss of expression of DNA repair proteins had a higher loss of cell control cycle.

## Introduction

Colorectal cancer (CRC), and gastric cancer (GC) are frequent cause of cancer worldwide. About 50% of individuals died from disease progression of CRC and 75% from GC (Bray et al., 2018). In Brazil, CRC is the third cause of cancer in men and the second in women and GC is the fourth cause of cancer in men and the fifth in women. (INCA,2018)

Colorectal carcinogenesis of sporadic tumors begins with the *APC* gene mutation, followed by mutations of the *KRAS*, *DCC *and *TP53* genes. The *APC* gene mutation promotes the formation of adenoma and decrease of β-catenin, mediator of the Wnt pathway that controls cell proliferation. Nowadays three pathways of carcinogenesis with different prognosis and therapeutic response are currently described (Collucci, 2013). The most common is the chromosome instability (CIS), the second is the microsatellite instability (MSI), common in hereditary CRC and in 15% of sporadic tumors and the hypermetylation of the CpG islands (CIMP) (Collucci, 2013).

Lynch syndrome (LS) is a hereditary syndrome with mutations of the mismatch repair genes (*MMR*), *MLH1*, *MSH2*, *MSH6* and *PMS2*. More recently, the deletion of the *EPCAM* gene has been included (Jass, 2007; Lynch et al., 2007). Carriers of LS had an increased risk to develop various types of cancers beyond CRC, such as endometrial, gastric, small intestine, ovary, hepatobiliary system and urinary tract (Jass, 2007).

During the repair process, MMR proteins form heterodimers, MLH1 matched to PMS2 and MSH2 matched to MSH6, so if there is a loss of MLH1 or MSH2, we will also have a loss of PMS2 or MSH6, respectively. Absence of MLH1 expression, may a consequence of mutation of BRAF (v600E) that cause hypermetylation of MLH1 and silence of the gene expression. The same can occur when the gene EPCAM is deleted and cause MSH2 silence (Ligtenberg et al., 2009) 

The suspicion of LS can be made by the positivity of the Amsterdam criteria I, later revised by the criteria of Amsterdam II and those of Bethesda. (Vasen et al., 1999; Umar et al., 2004; Lynch et al., 2007) 

The genes *p53*,* Cyclin D1*, *β-catenin*, *APC* and c-myc are oncogenes, tumor suppressor genes, and genes involved in the cell cycle phase. 

*TP53* is a tumor suppressor gene that acts on the cell cycle mechanism and on programmed cell death (apoptosis) (Lowe and Lin, 2000; Yildrim, 2015). Mutation of *p53* gene are described in 25% of adenomas and in 50-70% of patients with CRC (Qie and Diehl, 2016) GC studies suggest that patients with no p53 expression have a higher survival rate in relation to patients who have the mutation of this protein (Motokura et al., 2003 )

 Cyclin D1, also known as CCND1, is involved in cell cycle phase transition, this protein coordinates cell cycle progression with extracellular stimulation. Cancer cells had frequently defects in the G1/S phase, leading to unregulated growth, development and progression of the tumor (White et al., 2012; Luo et al., 2017)

The β-catenin protein acts on the Wnt signaling pathway and can induce the expression of the proteins cyclin-D1 and c-Myc (Thompson, 1998). Mutation in *APC* and *β-catenin* is present in more than 90% of CRC, thus highlighting the Wnt pathway. β-catenin can penetrate the nucleus and activate the transcription of the growth promoter genes (Conzen et al., 2000). 

C-myc is an oncogene usually found in molecular disorders that promote neoplasia. The protein is a nuclear phosphoprotein that stimulates the progress of the cell cycle, and apoptosis. C-myc also had a participation in the regulation of the apoptotic process (Forones et al., 2005; Zlobec et al., 2008) showing that both excess and loss of C-MYC expression can promote cell death. 

Carcinogenesis is a multifactorial process involving different proteins, mainly described in sporadic colorectal or gastric cancer. Studies of these proteins in tumor with loss of MMR proteins and positive criteria of Bethesda were not find in the literature.

The aim of the study was to evaluate the expression of p53, cyclin D1, β-catenin, APC and c-myc proteins in patients with colorectal cancer or gastric cancer with suspected of Lynch syndrome by Bethesda criteria and loss of expression of *MLH1, MSH2, MSH6 *and *PMS2*

**Figure 1:**
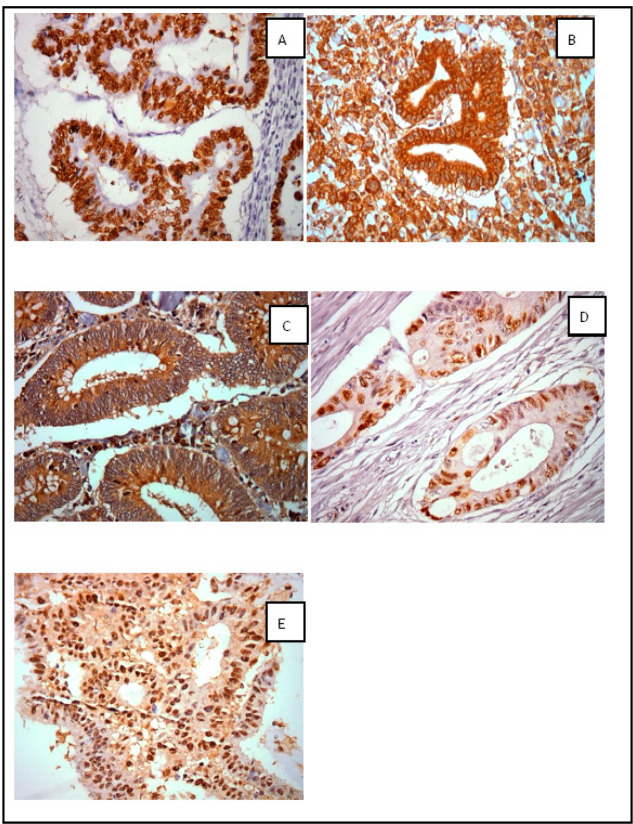
Photomicrographs. Positive nuclear immunoexpression of p53 (A), positive cytoplasmic and membranar immunoexpression and cytoplasmic β-catenin immunoexpression (B) positive cytoplasm immunoexpression of APC (C), positive nuclear immunoexpression of cyclin D1 (D) and positive nuclear immunoexpression of c-myc (E) in tumor tissue from patients with at least one of the Bethesda criteria. Increase: 400x

**Table 1:** Clinical Characteristics of Patients Included in the Study

Sex	CRC N (%)	GC N (%)
Female	30/62 (48.4)	3/8 (37.5)
Male	32/62 (51.6)	5/8 (62.5)
Age (years)		
≤ 50	33/62 (53.2)	6/8 (75)
> 50	29/62 (46.8)	2/8 (25)
Alcoholism		
Yes	30/61 (49.2)	3/8 (37.5)
No	31/61 (50.8)	5/8 (62.5)
Smokers		
Yes	21/59 (35.6)	4/8 (50)
No	38/59 (64.4)	4/8 (50)
Location		
Right colon	21/61 (34.4)	
Left colon + rectum	40/61 (65.6)	
Stage		
I	4/61 (6.6)	1/8 (12.5)
II	25/61(40.9)	4/8 (50)
III	28/61 (45.9)	3/8 (37.5)
IV	4/61 (66)	0 (0)
Treatment		
Chemotherapy	36/62 (58.1)	7/8 (87.5)
Radiotherapy	7/62 (11.3)	0 (0)
Combined	19/62 (30.6)	1/8 (12.5)
Follow up		
Alive	49/62 (79)	6/8 (75)
Death	10 /62 (16.1)	0/8 (0)
No information	3/62 (4.9)	2/8 (25)

**Table 2:** Negative Frequency of MLH1, MSH2, MSH6, PMS2 and Positivity of p53, β-catenin, APC, cyclin D1 and c-myc in Tumor Tissue

	CRC	GC
n negative / n total (%)		
MLH1	24/62 (38)	2/8 (25)
MSH2	11/62 (18)	1/8 (13)
MLH6	16/61 (26)	3/8 (38)
PMS2	30/62 (48)	6/8 (76)
n positive / n total (%)		
p53	55/62 (89)	6/8 (76)
Cyclin D1	32/58 (55)	2/7 (29)
Β-catenin	30/51 (59)	3/6 (50)
APC	34/57 (60)	3/7 (43)
c-myc	48/57 (84)	5/7 (71)

**Table 3:** Multiple Logistic Regression Model Used to Test Association with Negative MSH2 and PMS 2

		Estimate	Standard Error	Wald	p-value	OR	CI 95%	CI 95%
							Lower	Upper
Negative MSH2	+ Ciclina	0.953	0.435	4.786	0.029	6.73	1.22	37.16
Negative PMS2	Age	-0.07	0.033	4.41	0.036	0.933	0.874	0.995
	+ APC	0.734	0.364	4.07	0.044	4.34	1.043	18.063

+Ciclina, positive ciclina; +APC, positive APC; OR, odds ratio; CI, confidence interval

## Materials and Methods

Patients with colorectal or gastric adenocarcinoma with at least one of the criteria of Bethesda, over 18 years of age and, in follow up at the Gastrointestinal Oncology of the Universidade Federal de Sao Paulo were included. Patients with inadequate or insufficient quantities for immunohistochemical study or with a diagnosis of familial adenomatous polyposis or with inflammatory bowel disease were excluded.

This study was conducted according to the ethical standards and was approved by the Research Ethics Committee of the Universidade Federal de São Paulo.

Clinical and pathological data were analyzed retrospectively for all patients. 

*Immunohistochemistry*

The immunohistochemical technique for MLH1, MSH2, MSH6 and PMS2 was performed at the Laboratory of Diagnostic Immunohistochemistry of Pathology Department. The method consisted of EnVision FLEX +, Mouse, High pH (Link) and the primary antibodies clones were ES05, FE11, EP49 and PMS2 for MLH1, MSH2, MSh6 and PMS2 respectively from DAKO. The evaluation of the immunoexpression was performed according to the method proposed by Seo et al., (2003), with the classification of the results of the IHC tests as positive or negative. 

p53, β-catenin, APC, cyclin D1 were performed at the Laboratory of Experimental Molecular Pathology of the Pathology Department. The primary antibodies used in the research were FLEX mouse monoclonal anti-human-p53 (DO-7), FLEX mouse monoclonal anti-human beta-catenin (Beta-catenin -1) and FLEX rabbit monoclonal anti-human ciclin D1 (EP12) respectively from DAKO. The primary antibody for c-Myc and APC were anti-human c-myc mouse monoclonal antibody (9E10) and rabbit polyclonal antibody (C-20) respectively from Santa Cruz.

The immunohistochemical reactions were based on the streptavidin-biotin-peroxidase method. P53 and cyclin D1 were considered positive when it was expressed in more than 20% of the neoplastic nuclei (Lee et al., 2016), c-Myc in more than 10% of the neoplastic nuclei intensity (Therkildsen et al., 2012). β-catenin was analyzed in the cytoplasm and nucleus and APC in the cytoplasm, both were analyzed semiquantitatively using the scoring system based on the intensity of the reaction and the extent of staining.

*Statistical analysis*

 Data were analyzed statistically using Sigma Plot 11.0 software (Systat Software, Germany) and Statistica 13.2 software (Dell, Tulsa, USA), considering an alpha level of 5%. Normality was verified using the Kolmogorov-Smirnov test and the Quantile-quantile-plot (QQ-plot) graphs. The binomial regression was used to test the association between negative MLH1, MSH2, MLH6, PMS2 (dependent variables) and the others variables (gender, age, ethnicity, smoking status, degree of differentiation, tumor size and positivity for p53, β-catenin, APC, cyclin D1, and c-Myc)

After adjustment, two significant logistic regression models were obtained. Kaplan-Meier survival analysis and Log Rank test were used to determine whether the survival time distribution for events (death) differed based on MLH1, MSH2, MLH6, PMS2, p53, cyclin D1, β-catenin, APC and c-Myc. 

## Results

Seventy patients were included, being 62 with CRC and 8 with GC. Among the patients, we observed a predominance of males and patients under 50 years (55.7%). The mean age of the patients was 46.5 ± 11.4 years and the median were 43.5 years. More than half of the patients were nonalcoholic (52.2%) and no-smokers (62.7%). Pathologic stages II and III were predominant. In CRC, most of them was in the left colon (65.6%). The mean body mass index (BMI) was 25.9±5.2, 41.2% were eutrophic, 45.16% were overweight and 12.91% obese (Table 1). The photomicrographs of the immunohistochemical reactions of p53, APC, cyclin D1 and c-myc (Figure 1) 

We observed a higher prevalence of negative *p53* expression in tumors with absence of the immunoexpression of MLH1 protein, however these differences were not statistically significant. On the other hand, tumors with positive p53 immunoexpression was higher in tumors with the positive expression of MSH2. There was no difference in the expression of *cyclin D1*, *βcatenin*, *APC*, *c-MYC* in relation to the absence of immunoexpression of the MMR proteins (Table 2).

The result of the multiple logistic regression model used to test the association of the independent variables with the absence of MSH2 is presented in Table 3. A positive association between the absence of MSH2 and positive cyclin D1 was observed. In addition, the model showed that having cyclin D1 positive increases the chance of having MSH2 negative by 6-fold. The model explains 18.5% in the variation of MSH2.

We observed a negative association between the absence of PMS2 protein expression and age and a positive association between the absence of PMS2 and positive APC by the multiple logistic regression model used to test the association of the independent variables with the negative immunohistochemical result of PMS2 (Table 6). In addition, the model showed that with each year of age, there is a 7% reduction in the chance of having negative PMS2. Positive APC increases by 4 times the chance of having negative PMS2. The model explains 20% in the variation of PMS2.

We did not find difference in survival between the loss of the expression of the MMR proteins as well as for p53, cyclin D1, β-catenin, APC and c-MYC.

## Discussion

We observed in 67% of the patients with CRC or GC with positive criteria of Bethesda absence of *MLH1*, *MSH2*, *MSH6* and/or* PMS2* expression. For CRC, the results were 38.7% for MLH1, 17.7% for MSH2, 26.22% for MSLH6 and 48.38% for PMS2. In GC, there was a higher prevalence for MSH6 and PMS2 with 37.5% and 75%, respectively.

Colorectal and gastric carcinogenesis is a complex, gradual, multi-step process involving numerous factors, which can lead to several genetic alterations, including the mutation of certain critical genes, such as TP53, that accumulate during epithelial progression normal for carcinoma. The p53 protein, one of the most widely studied, is encoded by the *TP53* gene, having tumor suppression role, an important process in cell cycle regulation, repairing DNA damage, eliminating free radicals, regulating immune responses (Yildirim et al., 2015).

Immunohistochemical analysis of *p21*,* p27*, *p53* and *bcl-2* expression was performed using the microarray technique in a series of CRC tissues with preserved MLH1 and / or MSH2 and / or MSH6. The authors did not correlate these markers with prognosis (Zlobec et al., 2008). Wang et al., (2017) also performed a study on CRC tissue and showed that 58% of the tumors was p53 positive. Between 124 patients, 63 were men, mean age of 57.13 years and mean of BMI was 24.6. Among the 102 CRC, 22 was in the right colon, and most of them were well or moderately differentiate. The authors did not analyze the MMR proteins.

In our study, we observed *p53* expression in 88.7% of the samples, with no relation to any dependent or independent variable. Between the 70 patients, 62 had CRC and 8 GC, 70% were men, mainly among GC, the main age was 45.6 years, the majority were stages II or III and the BMI was 25.9, 58% were obese or overweight. The percent of younger patients in this study was probably due to be patients with Bethesda criteria. The frequency of p53 was higher in patients with positive expression of *MSH2*. Although this difference where not significant, probably tumors with chromosome instability had a higher risk of mutation of p53 than tumors with microsatellite instability. 

The study of cell proliferation and apoptosis in GC and intestinal metaplasia showed that p53 is commonly positive in tumors, mainly in advanced disease (68% in GC stage IV) (Zlobec et al., 2008). In our study, although the number of patients with GC was very short, p53 was positive in 75%. 

 Mutations and overexpression of *β-catenin* are associated with CRC and GC (MacDonald et al., 2009; Churillo, 2015). Dysregulation of Wnt/β-catenin Signaling in Gastrointestinal Cancers As a central molecule in the Wnt signaling pathway, β-catenin is in the membrane, cytoplasm and nucleus. Cytoplasmic and nuclear expressions are mainly involved in the regulation of the Wnt signaling path (Li et al., 2014). When the accumulation of β-catenin occurs in the nucleus there is activation of the E-cadherin, loss target gene expression program, linking the epithelial-mesenchymal transition to the Wnt signaling pathway (White et al., 2012).

In our series, 59% of CRC and 50% of GC tumors were positive for β-catenin. These numbers show that at least half of the patients had the Wnt / β-catenin signaling pathway activated.

The APC protein is also involved in the Wnt/β-catenin signaling pathway, forming with other proteins, a complex resulting in the degradation of β-catenin. When its expression is reduced by hypermethylation, allelic loss or mutation, it promotes a decrease in the degradation of β-catenin (Smith et al., 2002). *APC* mutation is commonly observed in CRC, but in GC the hypermethylation of the APC promoter gene is more frequent (Chiurillo et al., 2015). CRC and GC share the same spectrum of the* APC* gene mutation, exons 14 and 15 are the most frequently mutated region for both cancers (Smith et al, 2002; Fornasarig et al., 2018). Ghatak et al., (2017) have demonstrated novel *APC* gene changes in diffuse type of GC related to cell cycle abnormalities and expression of APC protein. Tunisian patients with GC had positive APC expression in 68.7%, β-catenin in 77.5% (Ayed-Guerfali et al., 2014). In the study by Bourroul et al., (2016) the immunoreactivity of the APC protein in CRC was 75% (Bourroul et al., 2016). Regarding the prognosis, overall survival time was significantly higher for patients with normal β-catenin expression alone or combined with positive *APC* expression. (Casimiro et al., 2012). The lower frequency of protein imunoexpression of APC in the cytoplasm, (59.6% in CRC tumors and 42.9% in GC), in our study, is probably a consequence of the patients included that had a LS suspicious, with negative expression of MMR protein.

The results of the multiple logistic regression, used to test the association of the independent variables with the immunohistochemical result of PMS2, showed a negative association between loss expression of *PMS2 *and age, because LS commonly affect younger patients. The positive association between absence of *PMS2* expression and positive APC, suggest that these patients had a higher index in mutation or deletion or methylation of the *APC* gene. Positive APC increases the chance of having absence of PMS2 in 4 times. 

Cyclin D1 is a protein that regulates the expression of cyclin-dependent kinases and function as a regulatory subunit of the cell cycle in the G1/S transition. Its overexpression can interrupt the control of the cell cycle allowing the development of cancer (Meyer and Penn, 2008). In CRC tumor tissues we found 55.2% nuclear positivity for cyclin D1 protein, whereas in GC it positivity is lower (28.6%). The nuclear expression was expected, since is a protein synthesized from the β-catenin stimulus in the cell nucleus, a finding that suggests that the canonical Wnt signaling pathway is activated. The results of the multiple logistic regression showed a positive association between loss of the imunoexpression of *MSH2 *and positive *cyclin D1*. A positive immunoexpression of *cyclin D1* increased the risk of absence of MSH2 by 6-fold. These results suggest that tumors with MSI had a higher loss of cell control cycle.

MYC or c-MYC is an essential cellular protein in the regulation of nucleotide metabolism, ribosome and protein synthesis, transcription and RNA processing and DNA replication. MYC regulates cell growth and proliferation. Mutations in the myc gene are found in lymphoma and carcinomas of the cervix, colon, rectum, breast, lung and stomach (Meyer and Penn, 2008).

Our results showed that 84.2% of CRC and 71.4% of GC had positive nuclear imunoexpression of c-myc. Lee et al., (2016) used immunohistochemistry, found a favorable prognosis when there was a co-expression of* c-myc* and *β-catenin* in CRC. This co-expression was independently correlated, suggesting that the expression of *c-myc* and *β-catenin* can be used as a predictive predictor of CRC patient prognosis. Toon et al., (2014), described a high percent of survival at 5 years in CRC that overexpress myc (93.2% vs. 57.3%). They concluded that the imunoexpression, determined by IHC, can be used to predict overall survival in patients with CRC undergoing surgical resection. In our study, we did not find correlation with this protein and survival.

The short number of patients and the absence of the results of the *MMR* genes sequencing are some limitations of the study. Currently little is known about the multiple interactions and mutual influences of the MMR proteins and the others involved in sporadic tumors which are necessary to promote tumor progression and malignancy.

In conclusion, the expression of β-catenin and c-MYC proteins in colorectal and gastric cancer tissues studied were independent of the immunoexpression of the repair proteins MLH1, MSH2, MSH6 and PMS2. Patients with loss of expression of PMS2 had a higher risk of mutation or deletion of APC and tumors with positive immunoexpression of *cyclin D1* had an increased risk of loss of expression of *MSH2*. These results suggest that tumors with MSI had a higher loss of cell control cycle.

## Funding statements

The study was supported by FAPESP Sao Paulo Research Foundation 14/21860.0 and CAPES Coordination for the Improvement of Higher Education Personnel.
